# The diagnostic journey of genetically defined neurodevelopmental disorders

**DOI:** 10.1186/s11689-022-09439-9

**Published:** 2022-05-02

**Authors:** Juliana Simon, Carly Hyde, Vidya Saravanapandian, Rujuta Wilson, Charlotte Distefano, Aaron Besterman, Shafali Jeste

**Affiliations:** 1 UCLA, 760 Westwood Plaza, Los Angeles, CA 90095 USA; 2 UCSD, 3020 Children’s Way, San Diego, CA 92123 USA; 3 CHLA, 4650 Sunset Blvd, MS #82, Los Angeles, CA 90027 USA

**Keywords:** Neurodevelopmental disorders, Genetic testing, Diagnostic journey

## Abstract

**Background:**

The development of advanced genetic technologies has resulted in rapid identification of genetic etiologies of neurodevelopmental disorders (NDDs) and has transformed the classification and diagnosis of various NDDs. However, diagnostic genetics has far outpaced our ability to provide timely medical counseling, guidance, and care for patients with genetically defined NDDs. These patients and their caregivers present with an unmet need for care coordination across multiple domains including medical, developmental, and psychiatric care and for educational resources and guidance from care professionals. After a genetic diagnosis is made, families also face several barriers in access to informed diagnostic evaluations and medical support.

**Methods:**

As part of Care and Research in Neurogenetics (CARING), a multidisciplinary clinical program for children and adults with neurogenetic disorders, we conducted qualitative clinical interviews about the diagnostic journey of families. This included the overall timeline to receiving diagnoses, experiences before and after diagnosis, barriers to care, and resources that helped them to navigate the diagnostic process.

**Results:**

A total of 37 interviews were conducted with parents of children ages 16 months to 33 years. Several key themes were identified: (1) delays between initial caregiver observations and formal developmental or genetic diagnoses; (2) practical barriers to clinical evaluation and care, including long wait times for an appointment, lack of insurance coverage, availability of local evaluations, transportation difficulties, and native language differences; (3) the importance of being part of a patient advocacy group to help navigate the diagnostic journey; and (4) unique challenges faced by adults (18 years or older).

**Conclusions:**

Families of children with complex neurodevelopmental and genetic disabilities face numerous challenges in finding adequate medical care and services for their child. They experience considerable delays in receiving timely diagnoses and face significant barriers that further delay the process of receiving access to services needed for the child’s continued care. The gaps indicated in this study speak to the need for more comprehensive coordination of care for patients with intellectual and developmental disabilities, as well as the development of systematic, disorder-specific resources both for providers and families in order to improve patient outcomes.

**Supplementary Information:**

The online version contains supplementary material available at 10.1186/s11689-022-09439-9.

## Background

Recent advances in genetic testing methodologies have helped guide our understanding of the neurobiology and phenotypic heterogeneity associated with neurodevelopmental disorders (NDDs). Chromosomal microarray and whole-exome sequencing have led to the identification of single-gene disorders and copy number variants associated with neurodevelopmental diagnoses, which include autism spectrum disorder (ASD), global developmental delay (GDD), intellectual disability (ID), and speech and language disorders [1, 2]. Although each genetic cause is individually rare, taken together, 15–53% of NDDs have an identifiable genetic etiology, depending on the specific presentation and test used [3–6].

For families of children with NDDs, early diagnosis and intervention are crucial to enhance quality of life [7]. Unfortunately, the precision of genetic diagnoses has not been matched with fastidiousness in clinical protocols to guide clinicians as they support patients and caregivers navigating the complex clinical and educational needs associated with these various conditions [8, 9]. Comprehensive counseling is essential in helping families make informed decisions regarding clinical care [10]. Caregivers of patients with NDDs often express skepticism when they are introduced to clinical genetic testing, as they may question its clinical utility [11]. However, the benefits of genetic testing have expanded and become far more tangible. For example, families can connect to one another through syndrome-specific patient advocacy groups (PAGs), expectations with regard to clinical sequelae and comorbidities can be provided, and targeted and disease-modifying therapeutics can now be identified [12, 13]. A starting point to improve clinical care of these neurogenetic disorders is to better understand the caregiver experience, particularly around the diagnostic journey, and then to evaluate the medical, educational, and service needs of the families.

As part of a comprehensive, multidisciplinary clinical program for children and adults with neurogenetic disorders, Care and Research in Neurogenetics (CARING), we conducted qualitative interviews about the diagnostic journey during clinical intake. Our goal was to capture the timeline and specific experiences around the genetic and neurodevelopmental diagnoses of patients and then to understand both challenges the families faced and helpful resources they received through the diagnostic journey. Through interviews with caregivers, we aimed to identify gaps in the diagnostic process and ensuing delivery of care that could directly guide the development of systematic, disorder-specific resources both for providers and families in order to improve outcomes for these patients with genetically defined NDDs.

## Methods

### Participants and data collection

**Fig. 1:**
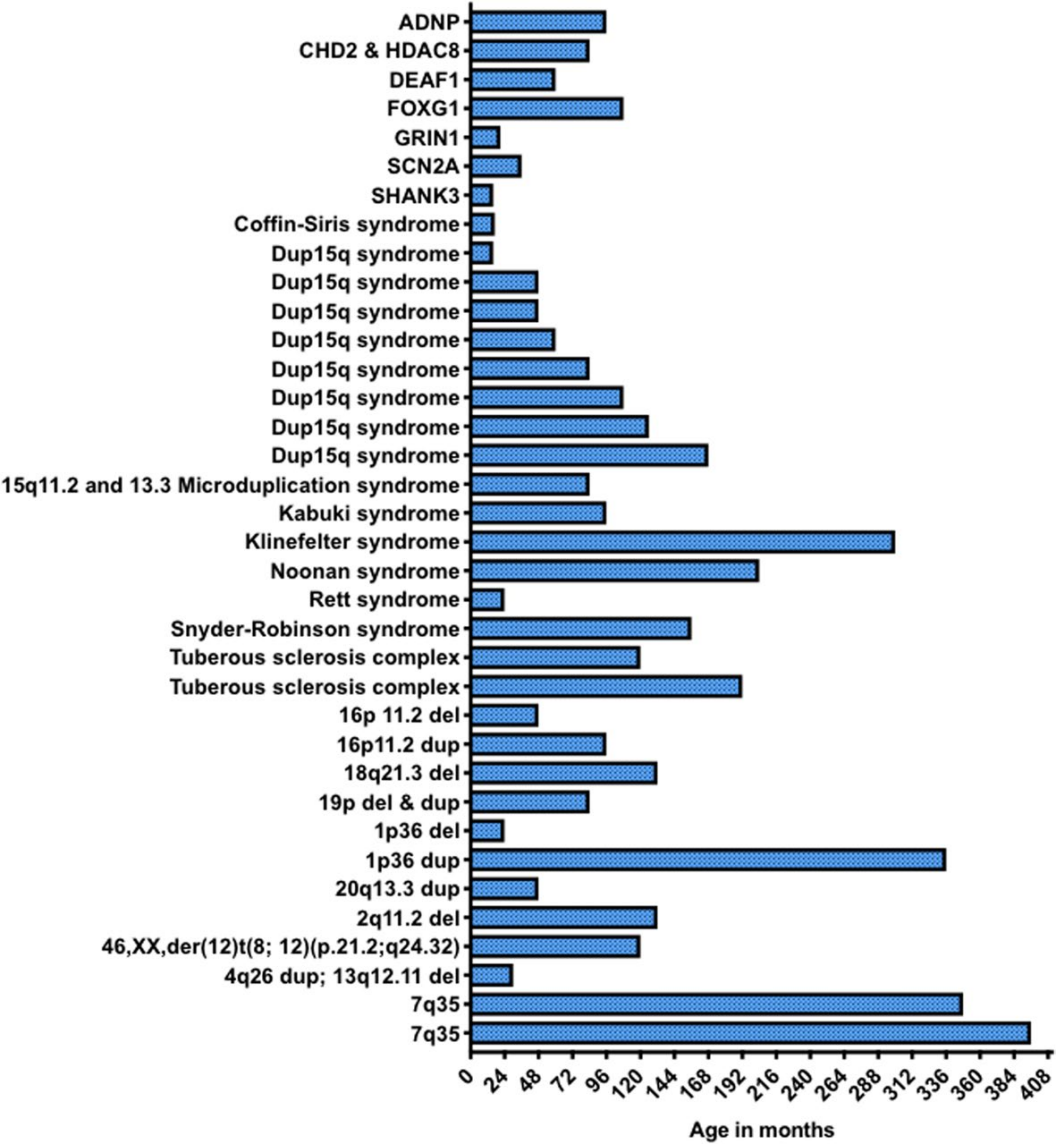
Age and genetic diagnosis of study participants

All caregivers of patients with confirmed neurogenetic diagnoses who had previously visited or had an upcoming appointment with the University of California, Los Angeles CARING clinic were eligible to share their family’s experiences for this study. Trained clinical administrators collected the diagnostic journey interview (DJI) by phone before (*n* = 15) or after (*n* = 22) the clinical appointment as part of the clinical triage (IRB#14-001908). Choice of timing of the interview was made based on caregiver availability, with the goal of minimizing burden on families. Interviews typically lasted 45–60 min, and responses were transcribed into the DJI form while the caregiver was interviewed. The DJI was administered to 37 families between 2017 and 2019. The children of the caregivers that participated ranged in age from 16 months to 33 years at the time of interview and represented 27 different genetically defined NDDs (Fig. 1).

### Diagnostic journey interview

Pilot interviews were conducted with three caregivers of children with genetically defined NDDs, and based on caregiver feedback, revisions were made to the content and structure of the DJI to include both multiple-choice and open-ended questions pertaining to all stages of the diagnostic journey. Questions in the DJI were categorized based on three major stages to diagnosis: (1) initial developmental concerns, (2) neurodevelopmental diagnosis, and (3) genetic diagnosis. Additionally, caregivers were asked to reflect on their experiences with the overall journey. The full questionnaire is provided in Additional file 1.

### Initial developmental concerns

Caregivers first were asked to discuss observations about their child’s developmental differences compared to peers, siblings, and their own expectations. Open-ended questions included age of their child at first developmental concern, type of first concerns (both developmental, medical, and other), time taken to seek medical advice, and type of provider evaluating their child (e.g., general practitioner, pediatrician, or other specialist). Caregivers were then asked to reflect on whether their child’s provider addressed their initial concerns, including acknowledging that the caregivers’ concerns were clinically valid or by providing further evaluation or support.

### Neurodevelopmental diagnosis

Caregivers were asked about their experiences with obtaining a formal neurodevelopmental diagnosis for their child following their initial concerns. Caregivers responded to open-ended questions about their child’s neurodevelopmental diagnosis (e.g., ASD, ID, GDD, or a speech and language disorder), including the child’s age at the time of diagnosis and the type of provider that made the diagnosis. Details regarding therapies and services (e.g., type of therapy, hours per week, and when services were first initiated) were collected in an effort to understand whether a new neurodevelopmental diagnosis affected access to services.

### Genetic diagnosis

This set of questions focused on experiences with genetic testing and counseling for the family following initial concerns. Open-ended questions pertaining to the family’s experience with genetic testing and counseling included the type of genetic testing, the type of diagnosis made, the type of provider ordering testing, and the information and counseling received following the diagnosis. Caregivers were then asked multiple-choice questions pertaining to the changes in medical care and access to services following the diagnosis.

### Appraisal of the overall journey

Caregivers were asked to evaluate their overall experiences throughout their child’s diagnostic journey. Caregivers indicated if certain practices were helpful, such as being part of a PAG or educating themselves by conducting their own Internet searches. Similarly, caregivers were asked about barriers that they faced in receiving necessary patient care (e.g., wait times for appointments, cost and availability of evaluations, insurance coverage) with “yes” or “no” responses. Finally, caregivers were asked open-ended questions that allowed them to reflect on their family’s experience navigating this diagnostic journey and to look ahead to the upcoming steps of care for their child.

### Data analysis

Interview transcripts were analyzed for common trends that described the trajectory of the diagnostic journey. Responses were coded and evaluated based on three major categories: (1) the individual’s diagnostic journey timeline, (2) barriers to patient care, and (3) key themes in the diagnostic experiences. Data pertaining to diagnostic journey experiences were descriptively analyzed throughout the entire collection process. Ongoing analysis of parent responses guided the identification of common trends across all interviews and allowed for qualitative description of the way families navigated the diagnostic journey process and its barriers. Due to the differences in specificity and scope of these diagnoses, we hypothesized that there could be differences in components of the diagnostic journey depending on whether a genetic diagnosis or a developmental diagnosis came first. Therefore, we separately considered individuals who first received a genetic and those who first received a developmental diagnosis. Excluded from this analysis were participants who received simultaneous neurodevelopmental and genetic diagnoses (*n* = 2), received genetic testing at time of birth due to immediate concerns at birth or in utero (*n* = 2), or did not provide the age of genetic diagnosis in the DJI (*n* = 3). Individuals who presented with concerns shortly after birth were still included and were marked as age 0 in our analysis. During data analysis, we also identified that four individuals (all over 18 years of age at the time of the interview) had experiences that were markedly different from the pediatric cohort. We hypothesized that younger children may benefit from the improved integration of genetic testing into clinical care, leading to less delays in the journey. Older individuals, in contrast, may have experienced unique delays in diagnosis due to lack of awareness or availability of genetic testing. Therefore, we separately quantified the experiences of families with children who were 18 years or older (*n* = 4) and those younger than 18 years (*n* = 33) at the time of the interview throughout analyses.

## Results

### Diagnostic journey timeline

From the interview, we identified four major phases of the diagnostic journey timeline: (1) time between first concerns and provider validation, (2) time between first concerns and developmental diagnosis, (3) time between first concerns and genetic diagnosis, and (4) time between genetic diagnosis and specialist referral. Average values for individuals younger than 18 years were computed and evaluated for each phase of the timeline. Individuals older than 18 years were analyzed separately. The following results depict the diagnostic journey timeline that precedes a developmental and genetic diagnosis for individuals younger than 18 in our study.

### First developmental concerns

For families of children younger than 18, age of first concerns ranged from birth to 60 months. In our sample, 58% of caregivers reported motor delays as the first concern, and 33% reported speech delays. First concerns were not always related to developmental observations, as 27% of caregivers reported medical issues (e.g., feeding issues, heart problems, seizures) as their first concern. Identified by 22% of caregivers, feeding concerns (e.g., issues with latching, vomiting, weight loss) within the first 6 months of life were the most common medical concern reported. Other first concerns included social communication delays (19%) and behavioral issues (15%).

#### Phase 1: first concerns and provider validation

On average, caregivers of individuals younger than 18 waited 5 months before their concerns were validated by a medical provider. Some caregivers reported immediate validation from their first provider, while others waited up to 2 years to find a medical provider who validated their initial concerns. One caregiver reported seeing four pediatricians within the first year due to lack of provider validation. Five caregivers indicated that medical providers attempted to assuage initial concerns, most notably when the child was younger than 12 months, by emphasizing the variability in developmental trajectories. As articulated by one caregiver, “We kept bringing up our concerns to the pediatrician at every baby check-up, and he kept advising we wait it out. He insisted that boys are slower sometimes, and that we didn’t need to be concerned so early on.”

#### Phase 2: first concerns and developmental diagnosis

The average duration from the caregiver’s first expressed concerns to formal neurodevelopmental diagnosis was 17 months, ranging from < 1 to 70 months for our participants younger than 18. One caregiver reported: “Our pediatrician did not take mental health seriously. No one was helping us even though our daughter was having outbursts and hurting people.” Caregivers noted that this lag in time resulted in an incurred financial burden as well. Without a formal diagnosis, families paid out of pocket for services. Discrepancies in diagnosis between providers accounted for another significant barrier. One caregiver reported that her child, who was initially diagnosed with ASD by a California Regional Center at 30 months, had her diagnosis changed at 36 months of age by the school district. She was subsequently denied services based on the new diagnosis alone.

#### Phase 3: first concerns and genetic diagnosis

Among participants younger than 18 years old, the lag in obtaining a genetic diagnosis averaged 30 months. Two individuals were immediately tested at birth due to either presentation of dysmorphic features or risk of a hereditary variant, while two others were tested shortly after due to significant medical concerns (e.g., seizures, heart dysfunction). The maximum wait time in our sample was 138 months. Families commonly struggled with insurance coverage for genetic testing, with one family reporting waiting over 3 years to receive genetic testing due to insurance authorization difficulties. Waitlists for appointments with geneticists and a lack of provider validation also led to delays in diagnosis at this phase. In cases where there was an existing neurodevelopmental diagnosis in place, some physicians did not recommend further genetic testing. In one case, a caregiver reported that her daughter’s neurologist did not believe there to be any genetic issues linked to her hypotonia and failure to thrive diagnoses, and she was advised to wait instead of seek testing. Caregivers reflected on the stress of navigating a child’s condition without a confirmed genetic diagnosis. As articulated by one caregiver, “It was eight years of agony not knowing what was wrong but knowing something wasn’t right.” Another caregiver commented on how receiving genetic testing results provided their family peace of mind: “It can only help you help your child more. It’s worth the initial fear and anxiety.”

#### Phase 4: genetic diagnosis and specialist referral

On average, families of children under 18 years old waited 3 months following their genetic diagnosis to meet with a specialist to discuss next steps in clinical management, with a range of 0 to 36 months. This was the shortest phase in the diagnostic timeline, as most families received the diagnosis from the ordering specialist. Therefore, the greatest barrier involved wait times for appointments with the referred specialist.

#### Phase comparison: evaluating timeline differences based on order of diagnosis

**Fig. 2:**
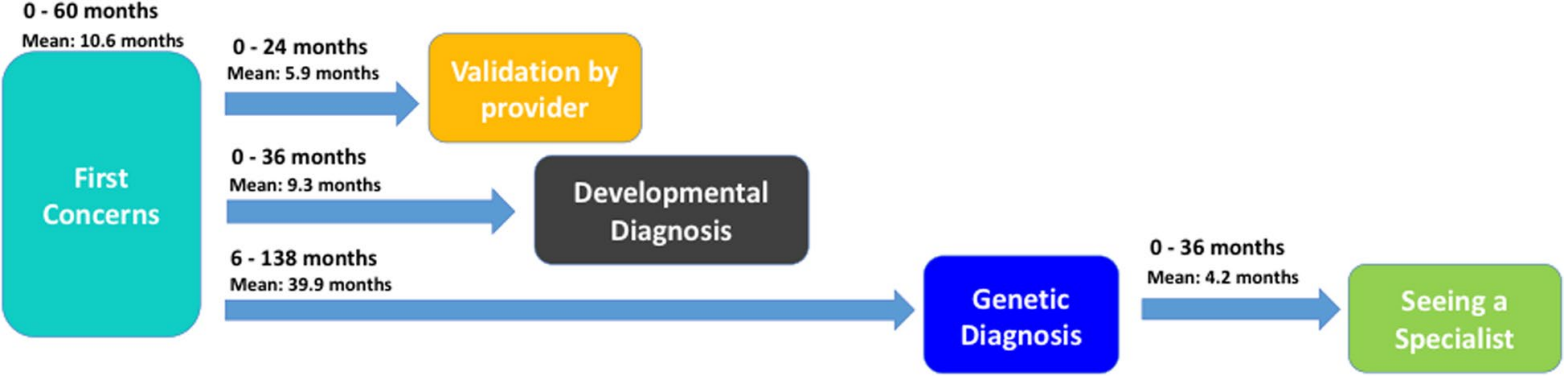
Diagnostic journey timeline for patients receiving developmental diagnosis before genetic diagnosis

**Fig. 3:**
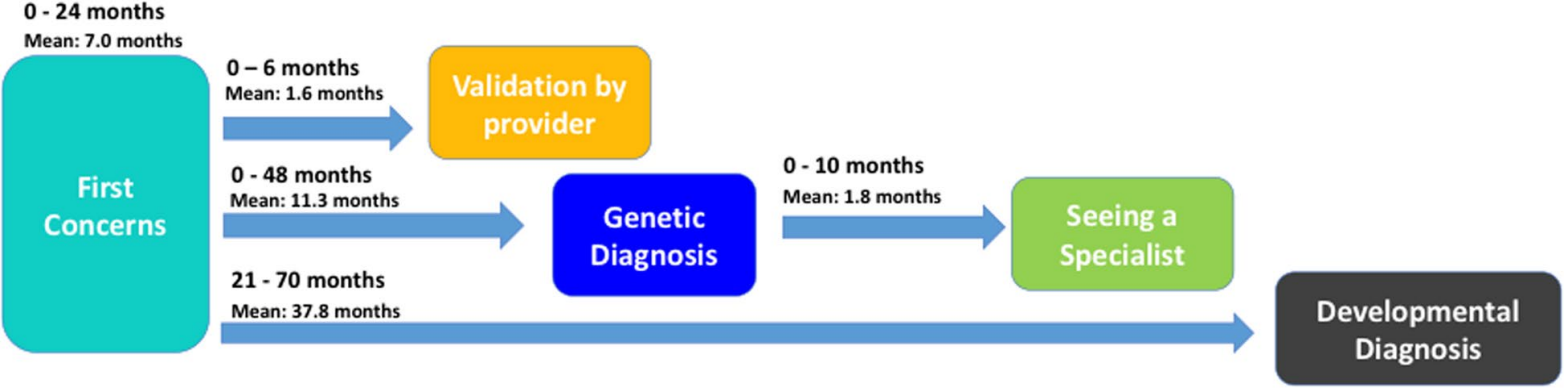
Diagnostic journey timeline for patients receiving genetic diagnosis before developmental diagnosis

The average values for each phase of the timeline were evaluated based on whether families received a developmental or genetic diagnosis first (Table 1). Two different timeline patterns described the general trajectory that families encountered based on whether they first received a genetic or developmental diagnosis (Figs. 2 and 3).

**Table 1:** Average values of diagnostic timeline compared between order of diagnosis

First diagnosis obtained	Age of first concerns (months)	First concerns and validation (months)	First concerns and developmental diagnosis (months)	First concerns and genetic diagnosis (months)	Genetic diagnosis and seeing a specialist (months)
**Developmental** (*n* = 18)	**10.6** Range: 0–60 SD: 14.4	**5.9** Range: 0–24 SD: 6.6	**9.3** Range: 0–36 SD: 8.8	**39.9** Range: 6–138 SD: 34.3	**4.2** Range: 0–36 SD: 10.1
**Genetic** (*n* = 8)	**7.0** Range: 0–24 SD: 7.6	**1.6** Range: 0–6 SD: 2.4	**37.8** Range: 21–70 SD: 19.2	**11.3** Range: 0–48 SD: 15.4	**1.8** Range: 0–10 SD: 3.6

The average gaps between key points of the journey were slightly shorter for the group who received a genetic diagnosis first. The combined time between receiving both neurodevelopmental and genetic diagnoses following initial concerns, however, was found to be similar in both groups, averaging approximately 4 years. Notably, the average amount of time elapsed between first concerns and developmental diagnosis for children who received a genetic diagnosis first was just over 3 years (37.8 months).

### Barriers to patient care

We assessed the types of barriers that all participating families experienced throughout the diagnostic journey. All caregivers interviewed (*n* = 37) were asked to evaluate via “yes” or “no” responses if certain barriers factored into their child’s diagnostic delays. A total of 59% of all caregivers (*n* = 22) reported wait time to meet with a specialist as a significant barrier to their diagnostic journey. A total of 49% of caregivers (*n* = 18) experienced problems with insurance. Some caregivers paid out of pocket for therapies and other health services that their child required, while others dealt with insurance authorization issues that significantly delayed genetic testing. A total of 35% of caregivers (*n* = 13) had difficulty accessing care in their area, and 41% of caregivers (*n* = 15) reported experiencing inadequate genetic counseling at the time of diagnosis. In cases where the ordering provider was a pediatrician or neurologist (*n* = 9), only one family was not appropriately referred to a genetic specialist after receiving their results. In some cases, caregivers reported that their physician lacked knowledge about their child’s specific genetic condition, often due to its rarity. One caregiver of a child with a *FOXG1* variant recalled her experience meeting with a geneticist. “The doctor gave us the results, then left for about an hour to do some research on the finding, as he admitted he was unprepared. He came back with a couple of case studies and apologized that he didn’t have more information to give.” Another caregiver similarly reflected on insufficient counseling, as her family was told very little information about the diagnosis and was further advised to “not read up too much on it” due to a lack of available knowledge on the disorder and prognosis.

### Self-advocacy

Caregivers emphasized the importance of self-advocacy while navigating the diagnostic journey, even when their own intuition may have been incongruous with physician expertise. When asked what type of advice they would give other families going through this process, caregivers stated: “Don’t take no for an answer,” and “Just keep fighting; don’t give up.” Other caregivers reported, “Be your child’s advocate and voice. Do your homework and don’t expect others to do it for you,” as well as, “Don’t assume that doctors or nurses know. Be assertive.”

### Patient advocacy groups

Among all participants, 76% of families (*n* = 28) reported PAG participation. Of those participating in a PAG, 68% of families (*n* = 19) indicated that they had confidence in how to proceed with the next steps in care for their child. In contrast, 44% of families who were not members of a PAG (*n* = 4) indicated confidence in next steps. Caregivers of children under 3 years of age reported the highest PAG participation and confidence in their next steps in clinical care across our sample (Table 2).

**Table 2:** Involvement in PAG and confidence in next steps across age groups

Age at time of interview (years)	Involvement in PAG	Good idea of next steps	PAG + good idea of next steps
**0–2** (*n* = 6)	100%	100%	100%
**3–4** (*n* = 6)	67%	50%	75%
**5–12** (*n* = 16)	81%	60%	62%
**13–18** (*n* = 4)	75%	50%	33%
**18+** (*n* = 4)	50%	50%	50%
**Total (*n*** **= 36)**	**76%**	**62%**	**68%**

Some families were unable to participate due to lack of an existing PAG specific to their child’s genetic condition. A few families chose not to participate in a PAG, reflecting that phenotypic variability, including variable symptomatology, could be discouraging. For example, one caregiver noted: “I wish I knew less. Parents tend to post everything.”

### Diagnosis and clinical care for adults

There were more delays in the diagnostic timelines for adults compared to the children (Table 3). On average, caregivers of adults reported waiting 261 months, almost 22 years, after voicing their initial concerns, before receiving a genetic diagnosis confirming their child’s condition. Families therefore had to navigate the logistical barriers associated with accessing services without a genetic diagnosis. For instance, families experienced issues with sustained funding for services provided by government-funded nonprofit agencies. All four caregivers similarly expressed disappointment in genetic counseling and overall management of their adult children. One caregiver reflected: “No one has a handle on what’s going on. There are all of these providers, but no answers.”

**Table 3:** Average values of diagnostic timeline compared across age groups

Age at time of interview (years)	Age of first concerns (months)	First concerns and validation (months)	First concerns and developmental diagnosis (months)	First concerns and genetic diagnosis (months)	Genetic diagnosis and seeing a specialist (months)
**0–2** (*n* = 6)	**3.5**	**1.7**	**2.5**	**9.5**	**0.7**
**3–4** (*n* = 6)	**8.3**	**5.8**	**15.9**	**13.8**	**0.2**
**5–12** (*n* = 16)	**8.0**	**6.0**	**20.6**	**32.1**	**2.6**
**13–18** (*n* = 4)	**23.3**	**4.5**	**18.0**	**64.5**	**12.0**
**18+** (*n* = 4)	**48.0**	**12.0**	**59.0**	**261.0**	**0.0**

## Discussion

Accurate and timely diagnoses of both genetic and neurodevelopmental diagnoses for children with NDDs are critical for families to access early intervention and support. Through the DJI, we sought to identify factors that defined the timeline and experiences of families of individuals with genetically defined neurodevelopmental disorders as they received diagnoses and adequate medical treatment and support. We also asked about the resources that helped them navigate their diagnostic journey. Our findings from the DJI highlight several key themes including the following: (1) delays between initial caregiver observations of developmental delays and formal developmental or genetic diagnoses, (2) multiple barriers to clinical evaluation and care, (3) the importance of being part of a PAG to help navigate the diagnostic journey, and (4) unique experiences faced by adults with NDDs.

This study does face some limitations. In our analysis, timing of interview in relation to clinical appointment (either before or after the CARING clinic appointment) was not considered as a significant variable, and this factor may have introduced some variability in caregiver perception of the overall journey and next steps to care. Additionally, we did not account for the effect of socioeconomic factors on the experiences of the participating families. We recognize the importance that SES holds in access to and quality of care, which would impact the overall journey and specific barriers to care, thus making this an important consideration for future research. Finally, our findings remain largely descriptive due to the small sample size. Expanding the number of participants would benefit the generalizability of our data. However, the detailed descriptions of the interviews and the trends identified are hypothesis generating and elucidate some critical themes that warrant further investigation and intervention.

The time span from concerns to diagnosis underscores the importance of including caregiver observations in early developmental screening and utilizing these observations to drive disorder-specific treatment plans for children and adults with NDDs. Caregivers often identify developmental concerns long before early symptoms might be observed or validated by a physician. Primary care physicians play a crucial role in delivering continuity of care, advocating access to expert clinicians, and providing psychological support. However, many caregivers in our study indicated that some physicians initially failed to acknowledge their concerns and were hesitant to take the necessary steps towards a timely evaluation. A lack of information and scientific knowledge about the constellation of symptoms associated with specific neurogenetic disorders may contribute to this issue [14]. Alerting primary care providers to developmental symptoms in a more systematic way may also help to reduce this gap. One possible approach is the implementation of digital medical toolboxes to provide caregivers with an accessible system to consistently document their concerns for physician reference, particularly through the integration of video recordings or standardized online checklists [15, 16]. Another option would involve creating a network of diverse specialists to share knowledge and resources among healthcare providers through teleconferencing and telementoring, an approach that has been operationalized for a variety of medical conditions through Project Extension for Community Healthcare Outcomes (Project ECHO) [17–20]. These approaches may help bridge the knowledge gap and bolster earlier physician validation of caregiver concerns [21].

When comparing diagnostic timelines across age groups, we found that younger children experienced shorter delays in care than older children (Table 3). This trajectory was especially notable in the age of first concerns and the lag between first concerns and genetic diagnosis across age groups. This difference may reflect increasing caregiver participation in community support groups, improving self-advocacy, and increasing caregiver awareness of developmental milestones, as well as physicians’ knowledge on the need for genetic testing.

Order of diagnosis was found to have a minimal effect on the diagnostic timeline in our sample of participants (Table 1), with both cohorts waiting approximately 4 years on average after initial concerns before receiving a neurodevelopmental and genetic diagnosis. Families that received a genetic diagnosis first only experienced marginally shorter timeline values throughout the journey, indicating a persistent gap between variant identification and clinical monitoring of patients with NDDs (Table 1). Although the average timeline values were similar for both groups, it is expected that the delay in diagnosis had differing consequences for individuals who received a genetic diagnosis first when compared to those who received a developmental diagnosis first. The lag between first concerns and developmental diagnosis following a genetic diagnosis leads to a delay in services and early intervention opportunities, which are critical for optimizing developmental gains. In contrast, for individuals that received a developmental diagnosis first, the lag between first concerns and genetic diagnosis limits diagnostic clarity and the ability to refer families to more focused patient advocacy groups, improve clinical monitoring, and recruit for clinical trials related to specific conditions. While these diagnostic gaps underscore the importance of listening to caregiver concerns, they may also indicate an issue with access to specialists that can make both developmental and genetic diagnoses.

With regard to the nature of the first concerns, we found that initial developmental concerns were most often centered around delayed motor milestones. Earliest signs of developmental delay are more likely to present in the motor domain for individuals with an NDD and an associated genetic variant [22, 23], suggesting that significant motor delay may increase the likelihood of a positive finding on genetic testing for patients with NDDs. It is important for physicians to be aware of this association, as it will allow for better prioritization of genetic testing and genetic referrals and allow for a more informed and nuanced discussion of the risks and benefit of genetic testing.

While physicians may feel inclined to assuage initial developmental concerns, especially in early infancy, it is important to identify which concerns are more commonly associated with genetic disorders, which would help accelerate diagnostic testing. Identification of significant motor delays as well as medical concerns associated with genetically defined NDDs can aid in this screening or risk stratification process. As corroborated in our results, concerns such as epilepsy, heart abnormalities, or facial dysmorphisms often prompt physicians to order genetic testing, resulting in expeditious care. However, in our sample, the most commonly reported medical concern pertained to feeding. Eight out of the ten caregivers who reported medical concerns cited feeding concerns and associated effects (e.g., vomiting and weight loss) within the first 6 months of life. Although feeding issues alone may prove insufficient for genetic testing, if they occur in conjunction with significant motor or social communication delays, further evaluation may be warranted. It must be emphasized that access to genetic testing and to an evaluation by a geneticist continue to be challenges that may undermine the diagnostic process, regardless of provider concern.

In fact, we found several practical barriers that precluded families from obtaining information and connecting to clinical resources, including problems with insurance coverage, lack of access to developmental experts in the area, wait time to get an appointment with a specialist, and inadequate genetic counseling. Improved physician education in genetics, reduced cost of genetic testing, and improved accessibility to clinical genetic experts could help address some of these current barriers. Hub-and-spoke models like ECHO may provide opportunities for dissemination of expertise. In this model, an expert “hub” provides consultation about patients to providers (“spokes”) through videoconferencing, empowering the local physicians to effectively care for complex patients [24, 25]. This process could minimize wait times for expert counseling, particularly for rare neurogenetic disorders. Moreover, creating multidisciplinary neurogenetics clinics, such as CARING, that support collaboration between clinical experts while providing comprehensive care to children and adults with NDDs will help with timely care and treatment.

Many families also reported significant difficulties connecting to critical treatment resources such as early intervention and therapies. While limited resource allocation may encourage insurance providers to require formal genetic and neurodevelopmental diagnoses prior to delivery of services, this barrier can cause a lengthy delay that can undermine timely access to care. High-quality intervention should be consistently available for children with NDDs regardless of formal genetic or neurodevelopmental diagnosis, as there likely are clear long-term benefits [26].

Parents that accessed PAGs reported more clarity around next steps in the journey. PAGs allow families to connect with one another and offer support through common experiences. They also provide families a platform to share useful resources regarding therapies or research opportunities, particularly those that may not be otherwise available due to existing barriers to care such as lack of timely diagnosis or not having access to clinical experts [27]. Collaboration between primary care physicians and PAGs may also aid in closing the gap between caregiver concerns and timely evaluation. PAGs also contribute to the success of the rare disease research by shaping research agendas, enhancing study feasibility and recruitment, and supporting training programs [28]. More than 70 PAGs actively participate in the Rare Diseases Clinical Research Network Steering Committee through the Coalition of Patient Advocacy Groups [28]. These successful collaborations help to bridge gaps between stakeholders and substantially impact research for rare diseases.

The small adult cohort in our sample provides a glimpse into the unique barriers that adults with NDDs encounter throughout the diagnostic process. Notably, initial developmental concerns were raised in the 1990s and the early 2000s for these individuals, marking a different era of medicine. When compared to the experiences of younger children, it is important to note how the current clinical management of NDDs has changed with the advancement and inclusion of precise genetic testing in clinical practice. Children under 2 years old at the time of the interview received both neurodevelopmental and genetic diagnoses within 1 year of caregivers first noting developmental concerns, suggesting that while advances in efficiency are still necessary, the diagnostic process has markedly improved over the last two decades. The significant diagnostic delays that adults faced posed even greater barriers to care and logistical challenges in accessing appropriate services that have since been ameliorated. In addition to these challenges that adults have faced, there is also a very limited natural history data for older patients with rare neurogenetic conditions. Clinical registries and natural history studies could improve our understanding of the clinical features of adults with these various genetic conditions. Therefore, it is imperative for clinicians to closely monitor these individuals in order to ensure appropriate clinical management moving forward, as well as establish a clearer understanding of how known genetic variants may affect symptomatology across the life span [29].

## Conclusions

Overall, our findings suggest that the diagnostic journey for individuals with genetically defined neurodevelopmental disorders is one that is long and often challenging. Providers can learn from caregiver experiences to improve their ability to support families throughout the journey. Caregivers face significant emotional, financial, and logistical burdens from searching for diagnostic answers while simultaneously caring for their child’s medical and developmental needs. Listening to the experiences of families navigating this journey is essential in addressing the specific barriers that preclude children and adults from receiving diagnoses and connecting to services. By identifying systematic delays in this process, clinicians can further work towards standardizing diagnostic recommendations. These qualitative data can facilitate the development of best practice guidelines in clinical care, enrich educational efforts for families and primary care providers, and encourage change in the clinical recommendations and counseling that families receive, ultimately helping to minimize the undue burdens that families currently face.

### Supplementary Information

**Additional file 1.** Diagnostic Journey Interview Guide.

## Data Availability

The datasets used and/or analyzed during the current study are available from the corresponding author on reasonable request.
