# Correction: The diagnostic journey of genetically defined neurodevelopmental disorders

**DOI:** 10.1186/s11689-023-09509-6

**Published:** 2023-11-11

**Authors:** Juliana Simon, Carly Hyde, Vidya Saravanapandian, Rujuta Wilson, Benjamin Schneider, Charlotte Distefano, Aaron Besterman, Shafali Jeste

**Affiliations:** 1 UCLA, 760 Westwood Plaza, Los Angeles, CA 90095 USA; 2 UCSD, 3020 Children’s Way, San Diego, CA 92123 USA; 3 CHLA, 4650 Sunset Blvd, MS #82, Los Angeles, CA 90027 USA

**Correction: J Neurodev Disord 14, 27 (2022)**

10.1186/s11689-022-09439-9

Following publication of the original article [1], the authors found that a contributor (Benjamin Schneider, UCLA, 760 Westwood Plaza, Los Angeles, CA, 90095, USA) was omitted from the publication authorship. The corrected authorship is shown in this article.
