# Supplementary material for: The diagnostic journey of genetically defined neurodevelopmental disorders
Source: J Neurodev Disord. 2022 May 2;14:27. doi: 10.1186/s11689-022-09439-9 (PMC9059413; doi:10.1186/s11689-022-09439-9)
Supplement: Supplementary file 1 — Additional file 1. Diagnostic Journey Interview Guide. [file 11689_2022_9439_MOESM1_ESM.pdf]

Diagnostic Journey Questionnaire

Interviewer:

Guardian completing the interview:

- o Mother
- o Father
- o Other ____________

First Concerns

How old was your child when you began to have concerns about his/her development, if any?

What were your concerns?

How long after you had these concerns did you see a medical provider about the concerns?

What type of provider did you see?

Prompts for interviewer:

- *General practitioner*
- *Pediatrician*
- *Other specialist (i.e. neurology, psychiatry, developmental pediatrician)*
- *Other (list): ______________________________*

What actions did this provider take?

Prompts for interviewer:

- *Conduct developmental evaluation*
- *Refer to specialist, specify:*
- *Refer for/recommend services*
    - o *Regional Center*
    - o *Early Intervention*
    - o *Public school system (IEP)*
- *Order additional testing*
    - o *Genetic testing*
    - o *MRI*
    - o *EEG*
    - o *Other, specify:*

Developmental Diagnosis

Was your child given a developmental diagnosis when you first expressed concerns and were evaluated?

What was that diagnosis?

Prompts:
- *Global Developmental Delay*
- *Intellectual disability*
- *Autism Spectrum Disorder/Aspergers/Pervasive Developmental Disorder*
- *Other developmental disorder (list here):* ____________________________

How old was your child when these diagnoses were made?

(If not already answered) Who made this diagnosis?

Did the first diagnosis result in the initiation of services or therapy?
If so, which services?

Following this diagnosis, did your child begin receiving any of the following services?

(1) Behavior interventions (e.g., ABA, floor time, PRT, CBT)	Yes No Don't Know<br><br>If so, please list type, how often and duration of each session:
(2) Speech therapy	Yes No Don't Know<br>If so, how often and duration of each session:
(3) Occupational therapy	Yes No Don't Know<br>If so, how often and duration of each session:
(4) Physical therapy	Yes No Don't Know<br>If so, how often and duration of each session:
(5) Special education	Yes No Don't Know<br>If so, how often and duration of each session:

(6) Early intervention	Yes No Don't Know<br>If so, how often and duration of each session:
(7) Other (please specify):	Yes No Don't Know<br>If so, how often and duration of each session:

Were these services provided through private insurance or at school?

How old was your child when he/she began receiving these services?

With new or changed developmental diagnoses, did services change? And how?

Child's Genetic Diagnosis

Does your child have a genetic diagnosis?
If so, what is it?

How old was your child when the genetic diagnosis was made?

Who ordered the genetic testing?

- Pediatrician/PCP
- Neurologist
- Geneticist
- Other:

What test was ordered?

Were you provided an explanation of your child's genetic test result by the ordering physician?

Did you receive formal genetic counseling (i.e., via a genetic counselor or geneticist) to discuss the results of the genetic test?
If so, what was explained to you?

How long after your child was given the genetic diagnosis did you first see a specialist with expertise in the disorder for treatment/care?

How did your child's medical care and therapies change after the genetic diagnosis was made? (check all that apply)?:

- Additional tests were ordered (specify):
- Started new treatments and therapies (specify):
- Increased the duration of therapies (specify):
- Nothing changed
- Other

Parent's Genetic Testing (if applicable)

Have either or both of the child's biological parents received genetic testing?

- Yes; if so, which tests were done?
- No
- I don't know

What was the genetic test result?

Using the scale below, select a number next to each statement to indicate how much you agree with the statement pertaining to *your child's* genetic diagnosis:

1 = strongly disagree
2 = disagree
3 = neither disagree nor agree
4 = agree
5 = strongly agree

I don't know if this genetic diagnosis could affect relatives (sisters, brothers, aunts, uncles, cousins).
1 2 3 4 5

In relation to my child's genetic diagnosis, nothing I decide will change my child's prognosis or development.
1 2 3 4 5

I know how to get the non-medical help my child needs (e.g., educational, social support).
1 2 3 4 5

I can explain what my child's diagnosis means to people outside my family who may need to know (e.g., teachers, social workers).
1 2 3 4 5

I don't know who else in my family might be at risk for this condition.
1 2 3 4 5

I feel guilty because I (might have) passed this condition on to my child.
1 2 3 4 5

Appraisal of overall odyssey

What kinds of things were the most helpful to you in getting help/obtaining information or services about your child's genetic diagnosis?

(1) Patient advocacy groups	Yes No Somewhat
(2) Social media (e.g., Facebook group)	Yes No Somewhat
(3) Professional organization (e.g., Autism Speaks, National Organization for Rare Disorders, Dup15q Alliance)	Yes No Somewhat
(4) Healthcare provider (specify)	Yes No Somewhat
(5) Independent internet searches	Yes No Somewhat

Were any of the following a problem with your experience of being diagnosed/referred/receiving services/accessing sufficient care?

	Developmental	Genetic
(1) Wait time to get an appointment?	Yes No	Yes No
(2) Cost of the evaluation?	Yes No	Yes No
(3) Availability of an evaluation in your area? (if so, how far did you travel to seek an evaluation?)	Yes No	Yes No
(4) Poor quality of the evaluation?	Yes No	Yes No
(5) Scheduling conflicts?	Yes No	Yes No
(6) Transportation?	Yes No	Yes No

(7) Insurance problems?	Yes No	Yes No
(8) Finding time to attend the appointment?	Yes No	Yes No
(9) Language barriers?	Yes No	Yes No
(10) Misunderstandings that may have resulted from cultural differences between you and the clinician?	Yes No	Yes No

Comments:

Since your child received a genetic diagnosis, have you reached out to other families or groups with the intention to share information or guide others with the same condition/ diagnosis?

1. Yes
2. No
3. Interested, but have not started
4. Not interested

If yes, which resources have been most useful (check all that apply)?

1. Social media (Facebook, etc.)
2. Websites specific to your child's condition
3. Family support groups
4. Meetings/alliances
5. Professional organizations
6. Medical staff/specialists
7. Patient advocacy groups
8. Other (please specify):

Have you sought any alternative treatments or therapies?

If you were to give advice to another family going through a similar diagnostic experience, what would you recommend?

Do you feel like you have the right team of doctors in place to cover your child's health care needs?

Do you feel like you have a good idea of next steps to take in this journey?
